# Evolution of Antibody Responses in HIV-1 CRF01_AE Acute Infection: Founder Envelope V1V2 Impacts the Timing and Magnitude of Autologous Neutralizing Antibodies

**DOI:** 10.1128/jvi.01635-22

**Published:** 2023-02-07

**Authors:** Syna Kuriakose Gift, Lindsay Wieczorek, Eric Sanders-Buell, Michelle Zemil, Sebastian Molnar, Gina Donofrio, Samantha Townsley, Agnes L. Chenine, Meera Bose, Hung V. Trinh, Brittani M. Barrows, Somchai Sriplienchan, Suchai Kitsiripornchai, Sorachai Nitayapan, Leigh-Anne Eller, Mangala Rao, Guido Ferrari, Nelson L. Michael, Julie A. Ake, Shelly J. Krebs, Merlin L. Robb, Sodsai Tovanabutra, Victoria R. Polonis

**Affiliations:** a U.S. Military HIV Research Program, Walter Reed Army Institute of Research, Silver Spring, Maryland, USA; b Henry M. Jackson Foundation for the Advancement of Military Medicine, Bethesda, Maryland, USA; c Department of Retrovirology, Armed Forces Research Institute of Medical Sciences, Bangkok, Thailand; d Royal Thai Army, Armed Forces Research Institute of Medical Sciences, Bangkok, Thailand; e Walter Reed Army Institute of Research, Silver Spring, Maryland, USA; f Human Vaccine Institute, Duke University School of Medicine, Durham, North Carolina, USA; g Department of Surgery, Duke University School of Medicine, Durham, North Carolina, USA; Emory University

**Keywords:** HIV-1, autologous neutralization, CRF01_AE, V1V2, ADCC, viral load set point, neutralization breadth

## Abstract

Understanding the dynamics of early immune responses to HIV-1 infection, including the evolution of initial neutralizing and antibody-dependent cellular cytotoxicity (ADCC)-mediating antibodies, will inform HIV vaccine design. In this study, we assess the development of autologous neutralizing antibodies (ANAbs) against founder envelopes (Envs) from 18 participants with HIV-1 CRF01_AE acute infection. The timing of ANAb development directly associated with the magnitude of the longitudinal ANAb response. Participants that developed ANAbs within 6 months of infection had significantly higher ANAb responses at 1 year (50% inhibitory concentration [IC_50_] geometric mean titer [GMT] = 2,010 versus 184; *P* = 0.001) and 2 years (GMT = 3,479 versus 340; *P* = 0.015), compared to participants that developed ANAb responses after 6 months. Participants with later development of ANAb tended to develop an earlier, potent heterologous tier 1 (92TH023) neutralizing antibody (NAb) response (*P* = 0.049). CRF01_AE founder Env V1V2 loop lengths correlated indirectly with the timing (*P* = 0.002, *r* = −0.675) and directly with magnitude (*P* = 0.005, *r* = 0.635) of ANAb responses; Envs with longer V1V2 loop lengths elicited earlier and more potent ANAb responses. While ANAb responses did not associate with viral load, the viral load set point correlated directly with neutralization of the heterologous 92TH023 strain (*P* = 0.007, *r* = 0.638). In contrast, a striking inverse correlation was observed between viral load set point and peak ADCC against heterologous 92TH023 Env strain (*P* = 0.0005, *r* = −0.738). These data indicate that specific antibody functions can be differentially related to viral load set point and may affect HIV-1 pathogenesis. Exploiting Env properties, such as V1V2 length, could facilitate development of subtype-specific vaccines that elicit more effective immune responses and improved protection.

**IMPORTANCE** Development of an effective HIV-1 vaccine will be facilitated by better understanding the dynamics between the founder virus and the early humoral responses. Variations between subtypes may influence the evolution of immune responses and should be considered as we strive to understand these dynamics. In this study, autologous founder envelope neutralization and heterologous functional humoral responses were evaluated after acute infection by HIV-1 CRF01_AE, a subtype that has not been thoroughly characterized. The evolution of these humoral responses was assessed in relation to envelope characteristics, magnitude of elicited immune responses, and viral load. Understanding immune parameters in natural infection will improve our understanding of protective responses and aid in the development of immunogens that elicit protective functional antibodies. Advancing our knowledge of correlates of positive clinical outcomes should lead to the design of more efficacious vaccines.

## INTRODUCTION

Immune responses in acute HIV-1 infection may affect disease features and progression. Understanding these events could contribute to improved vaccine design, as well as treatment approaches. Humoral immune responses to HIV-1 have been well characterized for subtype B, which is prominent in Europe, the Americas, and Oceania, and subtype C, which is prominent in Eastern and Southern Africa and India (1). However, understanding of other subtypes is not as well defined. Antibodies capable of neutralizing the founder subtype B and C viruses typically develop within months of infection (2, 3), while heterologous neutralizing antibodies (NAbs) appear after 1 or more years (4, 5). Of individuals who develop heterologous NAbs, only about 20 to 30% develop neutralization breadth (6, 7). While some studies have begun to focus on CRF01_AE infection and the developing immune responses (8, 9), a greater understanding of CRF01_AE founder Env properties and their elicited immune responses is needed to improve our understanding of humoral immunity in contemporary CRF01_AE infections.

CRF01_AE HIV-1, most prevalent in Asia, has Env features that appear to be distinct from other HIV-1 subtypes (1, 10, 11). The CRF01_AE Env is the least glycosylated among the subtypes, with an average of 28 potential *N*-linked glycosylation sites (12, 13). CRF01_AE Envs also contains an unusual histidine in the CD4-binding pocket at position 375, potentially leading to potentially greater sampling of the activated Env conformation (14). However, the incorporation of histidine at position 375 has not been shown to associate with Env antibody-dependent cellular cytotoxicity (ADCC) susceptibility, and these observations may vary by HIV-1 strain (15). This structural property has been implicated in the development of higher ADCC activity (16–19). Additionally, the neutralization sensitivities of CRF01_AE viruses are distinct from other subtypes (11). Further characterization is needed to understand how these unique properties of CRF01_AE HIV-1 Env affect the humoral immune response in natural infection and vaccination.

In the Thai RV144 vaccine trial, vaccination with CRF01_AE and subtype B antigens (canarypox vector ALVAC HIV [vCP1521] and AIDSVAX B/E gp120) lowered HIV acquisition by 31.2% (20). Analysis of this study showed a higher frequency of V2-specific antibodies in vaccinees (21), with an inverse correlation observed between V1V2-specific plasma IgG and infection risk (18, 22). Considering the potential protective role of V1V2-specific antibodies, a more in-depth evaluation of V1V2 antibody responses to CRF01_AE founder envelopes is warranted. V1V2 is a highly variable structure within Env (2, 23) with multiple *N*-linked glycans (24). This variable loop can shield neutralization sensitive epitopes within the Env trimeric spike (25–29) in a strain-specific manner (30, 31) and may facilitate immune evasion (29). Natural infection studies in subtype A and C have shown that V1V2-directed antibodies can contribute to autologous neutralization responses (32), with changes in the V1V2 loop resulting in neutralization resistance (32–35). Further understanding of V1V2-specific antibody responses in natural CRF01_AE HIV infection could inform modifications to vaccine antigens that may improve vaccine immunogenicity and elicitation of beneficial V1V2-specific antibodies.

The RV217 acute HIV-1 infection cohort is a prospective natural history study among high-risk participants from Thailand and East Africa (36). Healthy participants at increased risk for HIV acquisition were enrolled and initially monitored twice weekly. Upon detection of HIV-1 RNA from finger-stick blood samples, individuals were monitored frequently for 4 weeks and then at lengthening intervals for over 3 years. This study provided a detailed view of viral dynamics in acute infection. While other acute infection cohorts exist, these study samples are unique in their opportunity to evaluate the timing of acute CRF01_AE HIV-1 antibody development due to the close patient monitoring, defined seroconversion events, and frequency of sample collection after infection. The recently reported increase in CRF01_AE HIV prevalence, as well as the suggested faster clinical progression (37, 38), highlight the need for further investigation and continued vaccine development for this subtype.

In this study, we evaluated the longitudinal autologous and heterologous humoral immune responses to consensus founder Envs from 18 antiretroviral therapy (ART)-naive participants with CRF01_AE-infection. Here, we report specific distinctions among HIV-1 CRF01_AE founder Envs with respect to the magnitude and kinetics of antibodies that neutralize the autologous virus, NAb sensitivity, and ADCC activity and have investigated associations of these immune responses with viral load (VL) set point. Overall, HIV-1 CRF01_AE appears to take longer to induce autologous neutralizing antibodies (ANAbs) against the founder virus than has been previously reported for other subtypes (39). Participants with longer Env V1V2 lengths developed earlier autologous neutralizing antibodies, which reached higher magnitude over 3 years. A striking inverse correlation was seen between 92TH023 ADCC and VL set point, indicating plasma ADCC activity maybe be associated with improved viremic control. In four of these participants studied, the autologous NAb response preceded or coevolved with the autologous ADCC responses. Taken together, these data reveal insights into humoral responses that can inform HIV vaccine development for regions where CRF01_AE HIV-1 is prevalent.

## RESULTS

### Development of a Thai CRF01_AE founder Env pseudovirus panel.

Participants were selected for this study from the institutional review board (IRB)-approved, RV217 acute infection protocol executed in Thailand. Twelve male and six transgender female CRF01_AE HIV-infected participants were included; demographic data are available in Table S1. Founder *env* genes were cloned from plasma collected during the first week of diagnosed HIV infection. Of the 18 selected participants, 13 were infected with a single founder *env*, 4 participants were infected with 2 founder *env* genes, and 1 participant was infected with 5 founder *env* genes. *env* genes were cloned into expression vectors, and 26 founder HIV pseudoviruses (PSVs) were produced. All founder PSVs were determined to utilize the CCR5 coreceptor (data not shown), as typically seen in sexually transmitted HIV (40–42). Neutralization sensitivity of founder PSVs was evaluated using plasma from multisubtype plasma pools to determine neutralization tier phenotype, as previously established (43, 44). All founder PSVs were categorized as tier 2 (Fig. S1), corresponding to Env trimers that exist in a predominately closed conformation, except for the founder PSV from participant 217-B542219, which was categorized as tier 1b, corresponding to an intermediate Env conformation between closed and open (45).

### Development of antibodies capable of neutralizing autologous and heterologous HIV.

**FIG 1:**
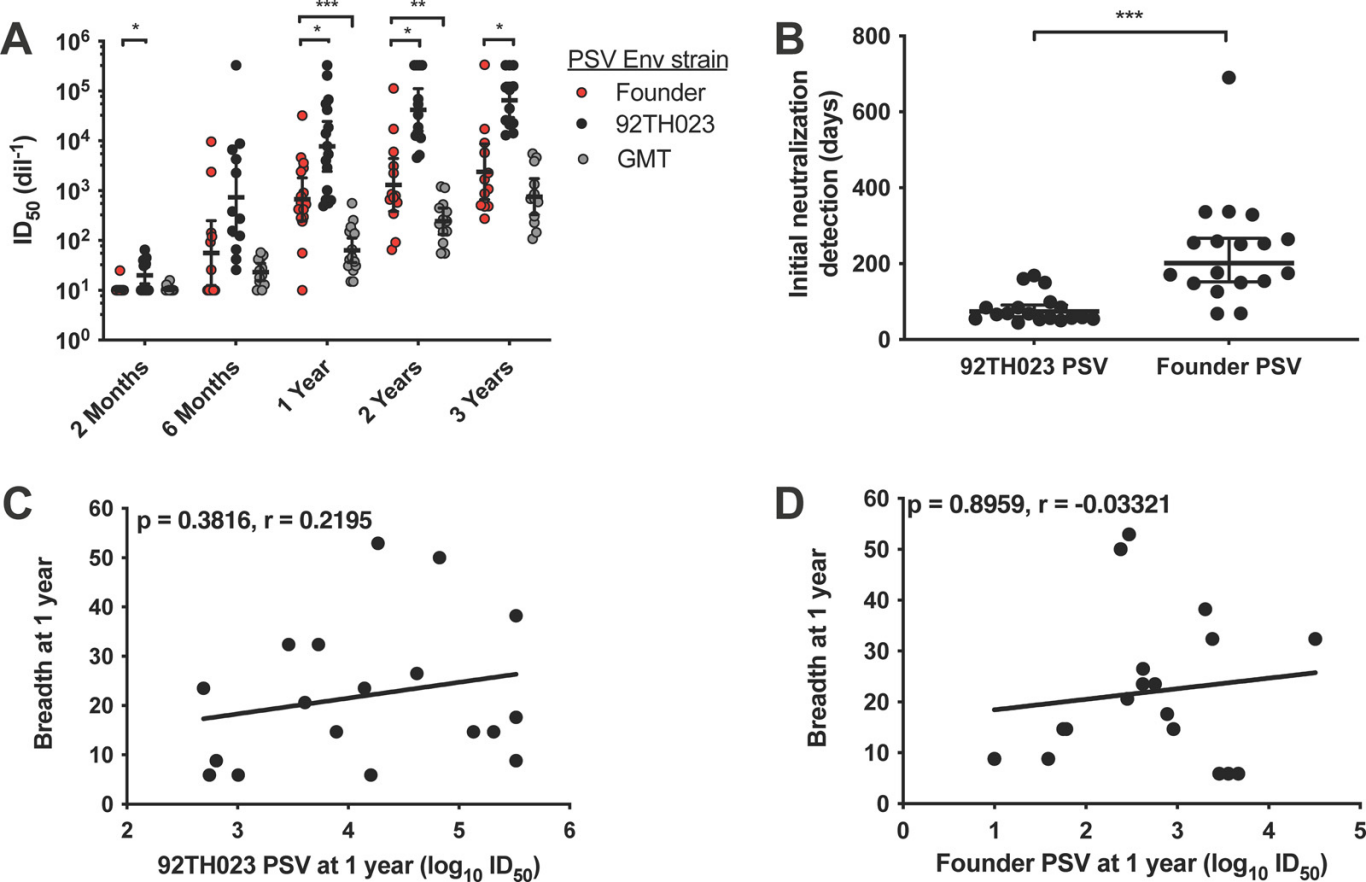
Longitudinal plasma neutralization of CRF01_AE founder viruses. (A) Plasma neutralization was evaluated using the autologous founder Env pseudovirus (PSV, red), heterologous tier 1 CRF01_AE 92TH023 PSV (black), and five tier 1 PSVs from subtypes A, B, and C and CRF01_AE that were used to generate a heterologous geometric mean titer (GMT, gray). Plasma neutralization was determined at multiple time points after infection; key time points are shown. (B) Time to detection of plasma neutralizing activity of the 92TH023 or autologous founder Env PSVs was determined. (C, D) The correlation between the magnitude of autologous founder Env PSV (C) or 92TH023 PSV (D), neutralization, and plasma neutralization breadth, determined using a panel of 35 diverse PSVs, was evaluated for plasma samples collected 1 year after infection. Significant differences between groups were determined by Mann-Whitney test and are indicated above the plots. *, *P* < 0.05; **, *P* < 0.005; ***, *P* < 0.0005. Correlations were evaluated by Spearman correlation analysis, and the trend lines are shown. Plasma with no neutralizing activity were given a 50% inhibitory dilution (ID_50_) value of 10.

Participant plasma samples collected longitudinally from preinfection up to 3 years postinfection were assessed for neutralization capacity against autologous founder and heterologous PSVs. Heterologous viruses included neutralization-sensitive (tier 1), CRF01_AE 92TH023, analyzed separately, and five tier 1 PSVs from subtypes A, B, and C and CRF01_AE that were used to generate a heterologous geometric mean titer (GMT). Plasma neutralization potency against autologous founder PSV, heterologous 92TH023 PSV, and heterologous PSV GMT at five time points is shown in Fig. 1A. Overall, plasma neutralization of the 92TH023 PSV (black dots) was observed earlier and at a higher magnitude over time than that observed for the autologous founder PSVs (Fig. 1A, red dots). Plasma neutralization of heterologous PSV GMT increased progressively over the 3-year period but was lower than both 92TH023 and autologous founder PSV neutralization in both timing and potency. Due to the frequency of plasma collections in the RV217 protocol, we were able to determine the timing of the development of autologous founder and heterologous 92TH023 PSV-neutralizing antibodies. Of the 18 participants, all had developed antibodies capable of neutralizing heterologous 92TH023 PSV by 6 months, yet only 9 participants were able to neutralize their autologous founder virus by 6 months (Fig. 1B). Antibodies capable of neutralizing heterologous 92TH023 PSV developed significantly earlier than antibodies capable of neutralizing the autologous founder PSV mean of 81 versus 234 days, respectively (Fig. 1B; *P* < 0.001).

To compare the development of autologous and heterologous neutralizing antibodies and the development of neutralization breadth in CRF01_AE infection, we compared the magnitude of autologous founder and 92TH023 PSV neutralization detected at 1 year with the neutralization breadth at 1 year, measured utilizing a panel of 35 diverse viral strains (7, 46). No association was observed between autologous founder (Fig. 1C) and heterologous 92TH023 (Fig. 1D) PSV neutralization and neutralization breadth at 1 year postinfection.

### Timing and potency of autologous neutralizing antibody development.

**FIG 2:**
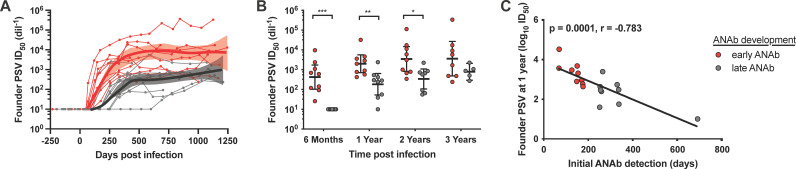
Timing and magnitude of plasma neutralization against the autologous founder Env pseudovirus (PSV). (A) Plasma neutralization of the autologous founder Env PSV was evaluated at multiple time points prior to and throughout infection for each participant; individual responses are shown as lines with evaluated time points marked with circles. Individuals that developed autologous neutralizing antibodies (ANAb) in the first 6 months (early ANAb) are shown in red; individuals that developed autologous neutralizing antibodies after more than 6 months (late ANAb) are shown in gray. Locally estimated scatterplot smoothing (LOESS) regression analysis and ribbons displaying 95% confidence interval was performed through R version 3.5.1. (B) The magnitude of autologous neutralization at key time points is shown for individuals that developed early (red) or late (gray) ANAb. (C) The correlation between the time to development of autologous neutralizing antibodies and their magnitude measured 1 year after infection was evaluated. Significant differences between groups were determined by Mann-Whitney test and are indicated above the plots. *, *P* < 0.05; **, *P* < 0.005; ***, *P* < 0.0005. Correlations were evaluated by Spearman correlation analysis, and the trend line is shown. Plasma samples with no neutralizing activity were given a 50% inhibitory dilution (ID_50_) value of 10.

We next differentiated participants by the timing to development of autologous founder PSV-neutralizing antibodies by utilizing the 6-month time point, at which time 9 participants had developed autologous neutralizing antibodies and 9 participants had not. Participants with early development of autologous neutralizing antibodies (<6 months, shown in red in Fig. 2) developed higher magnitude responses over time, compared to participants with later development of autologous neutralizing antibodies (>6 months; shown in gray in Fig. 2A). Participants with early autologous founder PSV neutralization developed neutralization capacity at a median of 150 days with a median peak plasma neutralization 50% inhibitory dilution (ID_50_) of 5,798. Plasma neutralization of 4 of these 9 participants exceeded an ID_50_ of 10,000. Participant 217-B542219, with the founder PSV tier 1b neutralization phenotype, had the most potent autologous neutralizing antibody response. Comparatively, participants with late autologous founder PSV neutralization developed neutralization capacity at a median of 264 days with a median peak neutralization ID_50_ of 860. Differences in the potency of autologous neutralization between these groups were significant at 6 months, 1 year, and 2 years (Fig. 2B; *P* < 0.0001, 0.001, and 0.015, respectively); fewer participants had plasma samples available at the 3-year time point due to anti-retroviral therapy (ART) initiation. A significant inverse correlation was observed between the time to development of autologous neutralizing antibodies and the potency of the autologous neutralizing antibodies measured at 1 year (Fig. 2C; *P* = 0.0001, *r* = −0.738). These data demonstrate that early development of antibodies capable of neutralizing the autologous founder virus results in more potent autologous neutralizing antibody responses over the course of infection.

We further evaluated the impact of multiple founder *env* genes on the timing to development of neutralizing antibodies. No significant difference was observed between the 5 participants with multiple founder *env* genes (median = 253 days, range = 150 to 337 days) and the 13 participants with single founder *env* genes (median = 175 days, range = 68 to 690 days; *P* = 0.503; data not shown). For participants with multiple founder *env* genes, the *env* with the earliest detectable plasma neutralization was included in Fig. 1 and 2 and all subsequent analyses.

### Neutralization sensitivity of CRF01_AE founder Env PSVs.

The neutralization sensitivity of the CRF01_AE founder PSVs to neutralizing monoclonal antibodies (MAbs) was then evaluated to identify potential Env features associated with autologous neutralizing antibody development. We utilized 20 neutralizing MAbs targeting the V1V2, V3, CD4-binding site (CD4bs), or the membrane proximal external region (MPER) of HIV-1 Env. The MAb geometric mean IC_50_ values are summarized in Table S2. Overall, the CRF01_AE founder Env PSVs were resistant to neutralization by b12 (CD4 bs), 2G12 (gp120 glycan), 447-52D (V3), and PGT151 (gp120-gp41 interface), and more than half of the PSVs were resistant to neutralization by sCD4 (CD4bs), PGT121 (V3), and Z13e1 (MPER).

**FIG 3:**
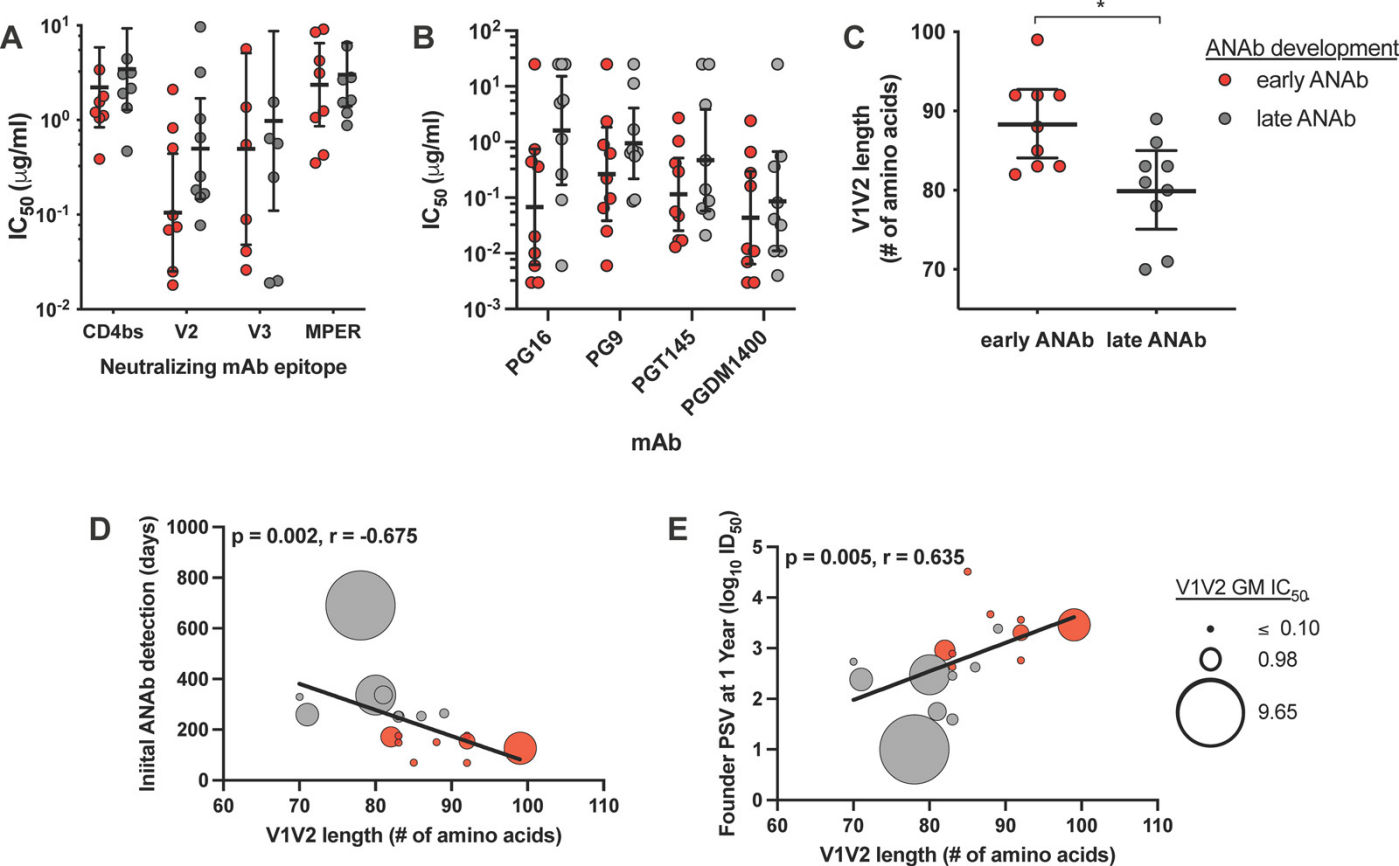
Founder Env features associated with autologous neutralizing antibody development. Neutralization sensitivity of founder Env PSVs was determined using a panel of monoclonal antibodies (MAbs) shown in Table S2. A founder Env pseudoviruses (PSVs) neutralization sensitivity score was calculated as the geometric mean of the 50% inhibitory concentration (IC_50_) values of MAbs targeting specific Env domains, either the CD4bs, V1V2, V3, or the membrane proximal external region (MPER). (A, B) Differences in founder Env PSV neutralization sensitivity by Env domain scores (A) or individual V1V2 MAbs (B) were evaluated between participants that developed early (red) or late (gray) autologous neutralizing antibody (ANAb) responses. (C) Founder Env V1V2 length was determined and compared between participants that developed early or late ANAb responses. (D, E) The correlation between founder Env V1V2 length and timing of ANAb development (D) or magnitude of the ANAb response 1 year after infection (E) was evaluated. For panels D and E, the colors of the symbols correspond to the categorization of participants by timing of ANAb detection: early (red) or late (gray). The sizes of the symbols correspond to the sensitivity of the founder Env PSV to neutralizing MAbs targeting the V1V2 Env domain. Significant differences between groups were determined by Mann-Whitney test and are indicated above the plots. *, *P* < 0.05. Correlations were evaluated by Spearman correlation analysis, and the trend lines are shown.

Participants that developed early autologous founder-neutralizing antibodies had Envs that trended toward higher sensitivity to neutralizing antibodies targeting the V1V2 loop, compared to participants that developed autologous neutralizing antibodies later in infection (Fig. 3A; *P* = 0.063). No significant differences were observed between groups in their founder Env PSV sensitivity to neutralizing antibodies targeting the CD4bs, V3, or MPER domains (Fig. 3A). Individual V1V2-specific antibodies included in this analysis are shown in Fig. 3B. While the differences between groups are not significant, and the data within each group are highly distributed, there is a trend toward greater V1V2 neutralization sensitivity for the founder Envs from individuals that developed early autologous neutralizing antibodies (Fig. 3B).

### Founder Env V1V2 length associated with development of autologous neutralizing antibodies.

The V1V2 loop length of the founder Envs was determined for comparison with the timing and magnitude of the elicited autologous neutralizing antibody response. Participants that developed early autologous neutralizing antibodies has significantly longer founder Env V1V2 loop lengths compared to participants that developed autologous neutralizing antibodies later in infection (Fig. 3C; *P* = 0.012). Founder Env V1V2 loop length also correlated indirectly with the timing of autologous neutralizing antibody development (Fig. 3D; *P* = 0.002, *r* = −0.675) and directly with the magnitude of autologous founder Env PSV neutralization measured at 1 year (Fig. 3E; *P* = 0.005, *r* = 0.635). Founder Envs with longer V1V2 loop lengths were generally more sensitive to neutralization by V1V2-specific neutralizing antibodies; as shown by the symbol size proportional to V1V2 IC_50_ magnitude in Fig. 3D and E. However, the correlation between V1V2 length and Env V1V2 neutralization sensitivity was not significant (*P* = 0.6754, *r* = −0.1060).

These data demonstrate that founder CRF01_AE Envs with longer V1V2 loops elicit more rapid and potent autologous neutralizing antibody responses. Founder Env V1V2 charge and the number of potential *N*-linked glycosylation sites did not correlate with the timing or magnitude of autologous neutralizing antibody responses (data not shown).

### Founder Env PSV V1V2 and V3 neutralization sensitivity associated with development of epitope-specific binding antibodies.

Participant plasma binding antibody responses to V1V2- and V3-specific antigens were evaluated to determine the relationship between autologous founder Env neutralization sensitivity and elicitation of epitope-specific antibody responses. Binding antibody data were available for a subset of participants from an analysis utilizing A244 gp70_V1V2 scaffold protein (gp70_V1V2) and 92TH023 cyclic V3 peptide (cV3) antigens (7). Founder Env PSV V1V2 or V3 neutralization sensitivity directly correlated with the timing to development of V1V2-specific (Fig. S2A) or V3-specific (Fig. S2B) binding antibody responses and indirectly correlated with the magnitude of the V1V2-specific (Fig. S2C) or V3-specific (Fig. S2D) binding antibody responses at 1 year, respectively. These correlations were significant for V3 responses, while the same trend was observed for V1V2 responses. These data indicate that increased neutralization sensitivity of founder CRF01_AE Env V1V2 and V3 domains tends to be associated with elicitation of more rapid and potent V1V2- and V3-specific binding antibody responses. However, no significant relationship was observed between V1V2- or V3-specific binding antibody responses and development of autologous neutralizing antibodies (data not shown). In addition, V1V2 length did not correlate with the timing or magnitude of the V1V2-binding antibody responses (data not shown).

### Development of ADCC activity in HIV-1 CRF01_AE infection.

Given the potential protective role of ADCC-mediating Abs in the Thai RV144 vaccine efficacy trial (19) and numerous studies demonstrating the protective effect of ADCC activity (47–49), we further evaluated development of ADCC-mediating antibodies in participant plasma. ADCC activity was measured for all participants using target cells infected with the heterologous CRF01_AE 92TH023 infectious molecular clone (IMC). Most participants (13 of 18) developed antibodies capable of mediating ADCC against the 92TH023 IMC within 6 months (data not shown). ADCC activity increased progressively over time; however, the timing and magnitude of ADCC activity did not correlate with the timing and magnitude of the neutralizing antibody activity (data not shown). Additionally, IMCs were constructed using founder Envs from four participants and were used to measure autologous plasma ADCC against those participants’ autologous founder Env.

**FIG 4:**
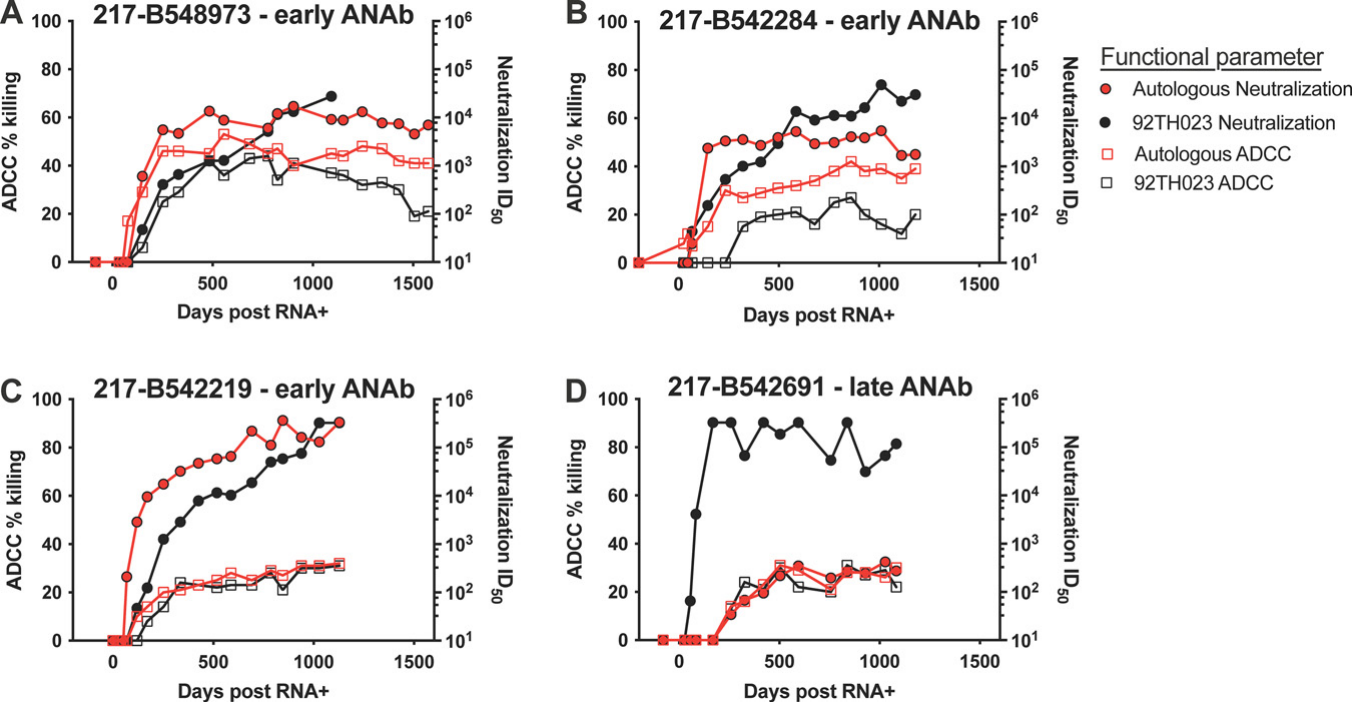
Development of neutralizing and non-neutralizing plasma antibodies. Longitudinal plasma antibody-dependent cellular cytotoxicity (ADCC) activity was evaluated using cell targets infected with the autologous founder Env or heterologous 92TH023 Env infectious molecular clone (IMC). Development of plasma neutralization and ADCC activity are shown for four participants, including three individuals that developed early autologous neutralizing antibody (ANAb): 217-B548973 (A), 217-B542284 (B), and 217-B542219 (C), and one individual that developed late ANAb: 217-B542691 (D). Plasma neutralization is shown with circle symbols; plasma ADCC is shown with square symbols. Activity against the autologous founder Env strain is shown with red symbols; activity against the heterologous 92TH023 Env strain is shown with black symbols.

Of the four selected participants, three developed early autologous neutralizing antibodies (217-B548973, 217-B542284, and 217-B542219), and one developed autologous neutralizing antibodies later in infection (217-B542691). Plasma ADCC against the 92TH023 and autologous Env IMCs were evaluated longitudinally for the four participants, as shown in Fig. 4. All three participants with early ANAbs (217-B548973, 217-B542219, and 217-B542284) developed antibodies capable of mediating ADCC against the IMC with the autologous Env prior to developing ADCC activity against the heterologous 92TH023 IMC (Fig. 4A to C). The participant with late ANAb development (217-B542691), showed an early, potent heterologous neutralizing antibody response to 92TH023 (Fig. 4D).

Because of this interesting observation regarding neutralization noted within these longitudinal data, we then assessed the full longitudinal neutralization profile of autologous founder Env PSV versus heterologous 92TH023 PSV neutralization for all 18 participants. Of the nine participants that developed autologous neutralizing antibodies later in infection, eight developed antibodies capable of neutralizing 92TH023 PSV earlier and more potently (by at least one log) compared to their autologous PSV neutralization (data not shown). However, this was only observed for three of the nine participants that developed early autologous neutralizing antibodies. This indicates a significant difference between the groups (Fisher’s exact test *P* = 0.049) in terms of the evolution of functional humoral responses to autologous versus tier 1 heterologous virus.

### Heterologous neutralization and ADCC responses associated with viral load set point.

**FIG 5:**
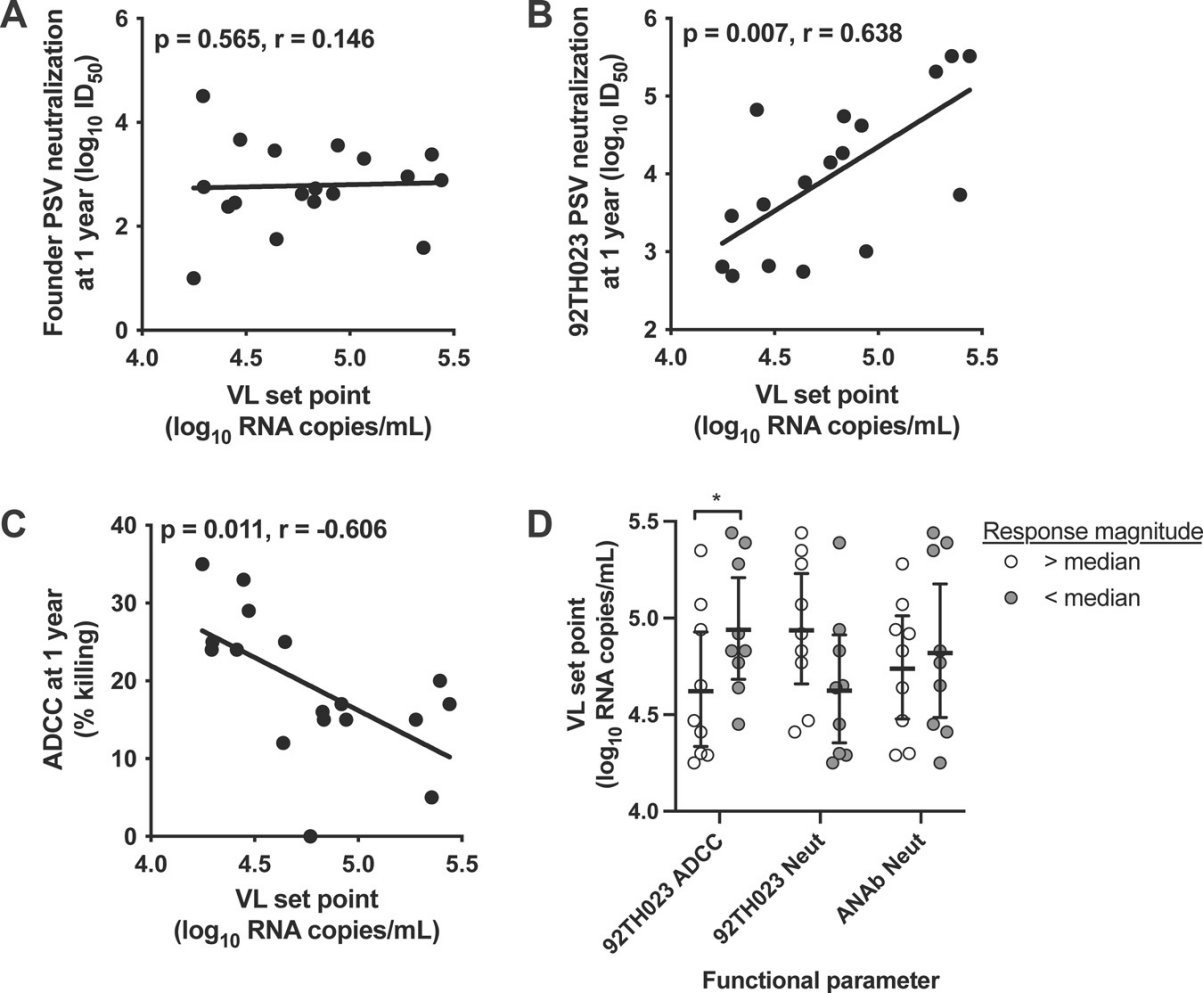
Relationship between humoral immune responses and viral load. The correlation was evaluated between participant’s set point viral load (VL). (A to C) Magnitude of autologous founder Env pseudovirus (PSV) neutralization (A), heterologous 92TH023 PSV neutralization (B), or heterologous 92TH023 IMC antibody-dependent cellular cytotoxicity (ADCC) activity (C) measured 1 year after infection. (D) Humoral immune responses were categorized as greater than (white) or less than (gray) the median value, and the set point viral load between groups was compared. Correlations were evaluated by Spearman correlation analysis, and the trend lines are shown.

The relationships between the participant’s clinical feature of viral load (VL) set point and their plasma antibody neutralization and ADCC functions were then analyzed to better understand the potential role of these immune responses in HIV disease. No correlation was observed between the autologous neutralizing antibody activity measured at 1 year and the VL set point (Fig. 5A). However, neutralization of the neutralization-sensitive (tier 1), heterologous CRF01_AE 92TH023 PSV strain measured at 1 year correlated directly with the VL set point (Fig. 5B; *P* = 0.007, *r* = 0.638). Importantly, a striking inverse correlation was also observed between the VL set point and ADCC activity measured at 1 year using the 92TH023 IMC (Fig. 5C; *P* = 0.011, *r* = −0.606). The peak of ADCC activity measured over 3 years showed an even stronger inverse correlation with VL set point (data not shown; *P* = 0.0005, *r* = −0.736). Plasma 92TH023 neutralization and ADCC activity measured at 1 year correlated inversely, but the trend was not significant (data not shown, *P* = 0.0835, *r* = −0.4329). We then compared plasma 92TH023 neutralization and ADCC activity to the plasma IgG subclass distribution for nine individuals for which these data were previously generated (Fig. S3). Neutralization activity correlated directly with CRF01_AE gp140-reactive plasma IgG1 (*P* = 0.006, and *r* = 0.850) and IgG4 (*P* = 0.040, *r* = 0.705), but not with IgG2 or IgG3. ADCC activity did not significantly correlate with CRF01_AE gp140-reactive plasma IgG1, IgG2, IgG3 or IgG4 (*P* value range from 0.119 to 0.904; data not shown).

Data endpoints were categorized as values above (white circles) or below (gray circles) the median response for each antibody function, and the VL set points were compared between the two groups with high or low functional antibody responses (Fig. 5D). Participants with ADCC activity above the median had lower VL set points. Higher VL set points tended to be observed in participants with higher levels of 92TH023 neutralization, while no difference was observed for autologous neutralizing antibody responses (Fig. 5D). Thus, participants with immune systems capable of mounting more potent ADCC responses tended to show better control of VL, while higher tier 1 NAbs may be an indicator of the increased antigenic exposure with higher VL.

## DISCUSSION

The RV217 cohort provides a unique opportunity to evaluate the development of humoral responses during acute CRF01_AE infection. Utilizing samples from this study, we wanted to determine whether features of the CRF01_AE founder Env could drive the development of functional antibodies, specifically focusing on earlier and higher magnitude neutralizing and ADCC-mediating antibody responses. We found that RV217 participants who developed ANAb responses within 6 months of infection also developed higher magnitude peak ANAb responses. These early responders maintained higher magnitude ANAbs longitudinally for up to 3 years, indicating that earlier responses lead to a greater overall potency of the ANAb response. An increase in antigen diversity during exposure through multiple infections did not result in earlier or greater ANAb titers. We observed a longer time to development of CRF01_AE founder ANAb responses (median of 31 weeks) compared to what has previously been reported for subtypes A (8 weeks) and C (14 weeks) (2, 3, 24, 50). Unfortunately, cross-subtype comparisons were not performed in this study to elucidate differences in humoral immune development against other subtypes. Underlying factors that can influence this outcome are currently being evaluated (7). Further studies are needed to understand the impact of early humoral responses on the evolution of the founder Env and development of neutralization breadth.

In this study, neutralization sensitivity of the CRF01_AE Env to V2 broadly neutralizing monoclonal antibodies (bNAbs) correlated with the development of earlier ANAb responses and V1V2-binding antibodies. A study evaluating Chinese CRF01_AE Envs (8) observed similar neutralization profiles as the Thai CRF01_AE founder Envs described here, including high sensitivity to some V2 bNAbs. The development of V1V2-specific antibodies has been implicated in mediating the first functional ANAb responses in subtype C (32). Analysis of the RV217 founder Env sequences revealed a significant relationship between longer V1V2 lengths and earlier development of ANAb responses, which was not previously observed for subtype C (33). Studies in subtype C infections have shown that shorter V1V2 loops lead to the development of neutralization breadth (34) and longer V1V2 loop lengths in subtype B coincided with increased neutralization resistance (29, 51). Also, in contrast to subtype C, we did not see a relationship between the number of V1V2 potential *N*-linked glycosylation sites (PNGSs) and ANAb titers (33). Many V2 bNAbs are dependent on glycosylations (52–54) and exert their effects by recognizing the quaternary structure of the V1V2 domains on the trimeric spike (52, 54–56); therefore, these subtype differences in neutralization could potentially be due to differences in Env glycosylation patterns between HIV subtypes. Our results indicate relationships between founder Env V1V2 epitope exposure and neutralization sensitivity during the development of ANAb responses that appear to be specific to CRF01_AE HIV.

In this study, neutralization of the heterologous 92TH023 HIV strain was observed prior to the detection of autologous neutralization of the founder Env, contrary to what has been previously reported for other subtypes (50). Prior vaccine studies have also shown that neutralization of tier 1 viruses, like 92TH023, is predominantly mediated by strain-specific, linear anti-V3 responses (31). In natural infection, these early V3-specific antibodies are not typically neutralizing due to V3 occlusion by V1V2 (57, 58). 92TH023 neutralization was detected as early as 2 months postinfection, and interestingly, individuals that developed earlier 92TH023 neutralization tended to develop later ANAb responses. This suggests a rapid development of decoy antibodies that may distract the immune system from development of more effective antibody responses over time (31, 57, 59–61). Similarly, V3 antibodies have been shown to develop early in subtype C infection, prior to the appearance of ANAb responses (median of 5 weeks versus 19 weeks), but they were not shown to contribute to ANAb responses (32, 33). We observed a correlation between founder Env sensitivity to V3-specific bNAbs and development of V3-binding antibodies, again indicating a relationship between founder Env epitope exposure and the specificity of antibodies elicited during natural CRF01_AE infection. Previous studies have indicated that the immune response to HIV-1 is driven by characteristics of the founder Env (62, 63), further demonstrating the importance of characterizing founder viruses. Additionally, a positive relationship between VL set point and neutralization of 92TH023 from 6 months through 3 years was observed, indicating that a higher antigenic load may lead to a greater magnitude of heterologous neutralization, as has been previously described (4, 5, 64–66).

HIV-1 VL set point has been used to evaluate the progression of HIV-1 disease (67–72) and vaccination efficacy during clinical trials (73) and during treatment-interruption studies (74, 75). Higher VL has been associated with increased mortality (71), while low VL or viremic control is a hallmark of those individuals designated long-term nonprogressors and elite controllers (76). The RV217 acute infection protocol is unique in the inclusion of high-risk participants prior to infection and in a sampling of participant plasma rapidly from very early time points, allowing for the determination of VL peak, nadir, and set point (36). With the availability of these data, we could begin to evaluate relationships between ANAb, heterologous virus neutralization, Env characteristics, and ADCC activity in association with VL set point. We observed an inverse correlation between lower VL set point and higher heterologous ADCC responses at 1 year and with peak ADCC activity observed over the course of infection; 92TH023 heterologous ADCC appears to be associated with viral control.

Interestingly, plasma neutralization of this same viral strain correlated directly with plasma VL. Individuals with higher viral loads have previously been shown to have higher plasma IgG and neutralizing antibody titers (4, 65). We similarly observed a significant positive correlation between plasma VL, neutralization titers, and Env-reactive IgG1. However, IgG levels or subclass did not associate with plasma ADCC activity, indicating that in part, different antibody populations may be mediating these two different plasma antibody functions. This discordance between plasma ADCC and neutralization has been previously demonstrated (15, 77, 78); this relationship may be affected by plasma potency, HIV-1 subtype, and target viral strain. Neutralizing antibodies have been shown to mediate ADCC activity (79), and ADCC-mediating non-neutralizing antibodies exist. Differences in plasma antibody ADCC and neutralizing activity may be observed due to differences in target Env expression between the two assays systems or through targeting of non-Env proteins in the ADCC assay. Additionally, while various ADCC assay formats have been shown to correlate, differences exist in the antigen availability for antibody recognition, as well as other variables (80). We used this particular ADCC assay format as we previously determined that it correlated with other relevant disease and vaccine outcomes (18, 81). However, use of another ADCC assay format could influence the trend observed in this study.

Previous studies have also shown inverse correlations between ADCC activity and VL (82–89). Another study evaluated ADCC activity during primary CRF01_AE HIV infection within a Chinese cohort and detected ADCC activity as early as 52 days postinfection, also noting an inverse correlation between ADCC activity and VL set point (9). ADCC was a potential correlate of protection from acquisition identified in the RV144 clinical trial (18). Studies in vaccinated rhesus macaques also demonstrate protection by ADCC-mediating antibodies and correlation with reduced acute viremia (90). ADCC-mediating antibodies have also been shown to inversely correlate with mother-to-child transmission among subtype A-infected women with high plasma VL (91). A larger collection of autologous founder Env proteins from the RV217 acute infection cohort is currently being evaluated for ADCC using protein-coated targets to delve deeper into the relationship between autologous ADCC and the development of neutralization and ADCC breadth.

The modest 31.2% efficacy of the RV144 vaccine trial highlights the importance of increasing vaccine immunogenicity and efficacy (18, 20). Due to significant genetic diversification of circulating HIV-1 strains observed over the last three decades (92, 93), there is a need to focus vaccine design on more contemporaneous Envs, such as these RV217 founder Envs, which were isolated between 2009 and 2015 (36). Additionally, the emergence of novel CRF01_AE recombinants (8, 94–96) and drug-resistant strains (97–100) has been reported. CRF01_AE infections appear to be rapidly expanding in certain countries and are now the dominant cause of new infections in China, Indonesia, Vietnam, and the Philippines (94, 101–105). Results from this study demonstrate that the humoral immune response to CRF01_AE HIV-1 infection is influenced by Env features, which may, in turn, affect viral load set point. While ANAb was directly related to V1V2 length and sensitivity to V2 MAbs, ANAb unfortunately showed no correlation with VL set point. With the production of autologous envelope proteins and more autologous IMC from the RV217 acute infection cohort, it will be important to study the relationships between autologous ADCC, V1V2 properties, and viral load control. Design of subtype-specific Env vaccine antigens with well defined features that can be related to viral control may then be used to elicit favorable humoral immune responses via vaccination to provide improved protection against HIV infection.

## MATERIALS AND METHODS

### Ethics statement.

For study RV217/WRAIR#1373 (FWA00000373 and IRB00000794) and study RV229/WRAIR#1386 (source of HIV-negative leukopaks for peripheral blood mononuclear cells [PBMCs] used as effector cells in ADCC assays), all participants were adults and provided written consent. All studies were reviewed and approved by the human ethics and safety committees in each country, as well as by the IRB of Walter Reed Army Institute of Research (Silver Spring, MD, USA), in compliance with all relevant federal guidelines and institutional policies. The human experimentation guidelines of the U.S. Army Surgeon General’s Human Subjects Research Review Board, the ethics review committee of the Ministry of Public Health of Thailand, and the institutional review boards of the Royal Thai Army Medical Department and of Mahidol University (FWA00001813 and IRB00001439) were followed in the conduct of this clinical research.

### Production of cohort *env* clones.

Genotyping and single genome amplification were performed as previously described (106, 107) to determine *env* sequence and subtype. Founder *env* sequences were isolated and analyzed from plasma samples taken within 1 week after HIV detection. Viral RNA was isolated using the QIAamp viral RNA minikit (Qiagen, Hilden, Germany) and was converted to cDNA through nested reverse transcription (RT)-PCR using the SuperScript III first-strand synthesis system (Invitrogen, Waltham, MA, USA). The *env* genes were amplified by nested PCR and then were sequenced using an ABI 3730xl DNA analyzer (Applied Biosystems, Waltham, MA, USA). Genotyping of HIV-1 viruses were performed as described previously by quantitative PCR amplification (92, 108). *env* genes were amplified with Platinum *Taq* polymerase (Invitrogen, Waltham, MA, USA); the PCR products were qualified through gel electrophoresis, extracted, and column-purified using the Macherey-Nagel nucleic acid purification kit (Clontech, Kyoto, Japan). The purified PCR products were then ligated into the pcDNA 3.1/V5-His TOPO TA (Invitrogen, Waltham, MA, USA) using the manufacturer’s recommendations. The ligation products were transformed into STBL4 cells (Invitrogen, Waltham, MA, USA), DNA clones with the correct insert were purified using the PureYield plasmid miniprep kit (Promega, Madison, WI, USA), and infectivity was screened using TZM-bl cells. The sequences were verified using an ABI 3730xl DNA sequencer (Applied Biosystems, Waltham, MA, USA). *env* genes with minimal mutations were mutated back to the consensus using QuikChange XL site-directed mutagenesis kit (Agilent, Santa Clara, CA, USA), or this was performed commercially (GeneWiz, Chelmsford, MA, USA). Two *env* genes, 217-B544996.F1 and 217-B547460.F1, were commercially synthesized and ligated into the pcDNA 3.1 vector (GenScript, Piscataway, NJ, USA).

### Virus stock production and titration.

Viruses were produced by transfection of HEK 293T cells. For PSV production, *env* DNA (8 μg) and pSG3Δenv (24 μg) were combined with X-tremeGENE 9 DNA transfection reagent (Sigma-Aldrich, St. Louis, MO, USA) at a 1:3 ratio as per the manufacturer’s recommendations, in serum-free Dulbecco’s modified Eagle’s medium (DMEM). For chimeric infectious molecular clone (CHIMC) production, 15 μg of plasmid DNA were combined with FuGENE 6 (Promega, Madison, WI, USA) at a 1:4 ratio as per manufacturer recommendations in serum-free DMEM. The transfection mix was incubated with the cells for 6 h at 37°C, and then the medium was replaced. Virus supernatants were harvested 72 h after transfection, filtered through a 0.22-μm PES membrane (Millipore, Burlington, MA, USA), and stored in 3-mL aliquots at −80°C. Viruses were titrated on TZM-bl cells in an endpoint dilution assay. Fourfold serial dilutions of PSV were added to 96-well black-walled plates (Corning, Corning, NY, USA) in a final volume of 25 μL/well. An additional 25 μL of supplemented DMEM was added to each well. TZM-bl cells were added to plates in 50 μL of supplemented DMEM with 40 μg/mL of DEAE-dextran (Sigma, St. Louis, MO, USA). Virus and cells were incubated together for 48 h at 37°C. Infectivity was measured by luminescence after adding 100 μL of the Bright-Glo luciferase assay system (Promega, Madison, WI, USA) to each well; luminescence was quantified using the Envision luminometer (Perkin Elmer, Waltham, MA, USA).

### GHOST cell assays.

The GHOST cell infection assay was used to determine coreceptor usage of viral stocks. Parental, CXCR4-expressing, or CCR5-expressing GHOST cells were cultured in 24-well plates. The cells were infected with undiluted PSV in the presence of 20 μg/mL of Polybrene infection reagent (Millipore-Sigma, Burlington, MA, USA) for 4 h. The cells were washed, cultured for 2 days, and then harvested and fixed with 2% paraformaldehyde. The percentage of infected cells expressing green fluorescent protein (GFP) was measured by flow cytometry using a LSRII cytometer and FACSDIVA software (Becton, Dickinson, Franklin Lakes, NJ, USA). Postgating analysis was conducted with FlowJo software (FlowJo, LLC, Ashland, OR, USA). PSVs were designated using the CCR5 or CXCR4 receptor if the percentage of GFP-positive cells for either the CCR5- or CXCR4- cell lines was 5-fold greater than the percentage of GFP-positive cells in the parental line. Assay controls included an uninfected negative-control, murine leukemia virus (MuLV) as a positive control for all lines and BaL.01 (CCR5-tropic) and MN.3 (CXCR4-tropic) PSVs as specific cell line controls.

### Antibodies, proteins, and plasma.

The following reagents were obtained through the National Institutes of Health (NIH) AIDS Reagent Program, Division of AIDS, NIAID, NIH: soluble CD4 recombinant protein (sCD4) from Progenics; VRC01 from John Mascola; 3BNC117 from Michel C. Nussenzweig; 10E8 from Mark Connors; PGT121, PGT126, and PGT145 from IAVI; and HIV-IG from NABI and NHLBI. B12, 2G12, PG9, PG16, 2F5, 447-52D, 4E10, and Z13e1 were purchased from Polymun. PGDM1400, PGT130, and PGT151 were graciously provided by Dennis Burton and Devin Sok at IAVI. VRC42.01 was generously provided by John Mascola and Nicole Doria-Rose at the Vaccine Research Center, NIH. PGT128 was made in-house by Vincent Dussupt. Anti-HIV-1 gp120 MAbs 697-30D was generously provided by Susan Zolla-Pazner, and anti-RSV was purchased from MedImmune (Frederick, MD, USA). Recombinant proteins (AE A244 gp70V1V2) utilized for surface plasmon resonance (SPR; Biacore, Uppsala, Sweden) were obtained from Bart Haynes and Hua-Xin Liao (Duke Protein Production Facility). RV217 study samples, viral load, and cell count data were collected as described by Robb et al. (36). Plasma pools A, B, C, D, CRF01_AE, CRF02_AG, and 21.2B (subtype B) and uninfected normal human sera were generated as described by Brown et al. (12).

### Virus neutralization.

Virus neutralization assays were performed to test plasma and MAb neutralization of PSVs. Plasma samples were prepared by heat inactivation at 56°C for 45 min, followed by 5-min centrifugation at 2,000 rpm to remove particulates. Plasma samples were used at a starting dilution of 1:20 in supplemented DMEM; MAbs were used at a starting concentration of 25 μg/mL in supplemented DMEM. Viruses were diluted to a concentration to yield luminescence at least three times above the cell-only controls, as determined by the PSV titration. Neutralization reagents were serially diluted 1:4 in independent duplicates per plate and incubated with equal volumes of virus for 1 h at 37°C before adding TZM-bl cells with 40 μg/mL of DEAE-dextran (Sigma, St. Louis, MO, USA). The plates were incubated at 37°C for 48 h before reading the luminescence. Nonspecific neutralization by plasma was evaluated using MuLV *env* DNA pseudotyped with the pSG3Δenv backbone DNA. Uninfected normal human serum was also tested against all the PSVs as a negative control.

### Antibody-dependent cellular cytotoxicity studies.

ADCC experiments were performed using CEM.NKR-CCR5+LUC+ target cells, as described previously (109). The 217-B548973, 217-B542284, 217-B542691, and 217-B542219 *env* genes were cloned into the CRF01_AE 40061. LucR backbone as described previously (110). The 40061.LucR_TH023Env CHIMC was then optimized for infection of CEM.NKR-CCR5+LUC+ target cells with 5 μg/mL DEAE-dextran (Sigma-Aldrich, St. Louis, MO, USA). 40061.LucR_217-B548973.F1env, 40061.LucR_217-B542284.F1env, 40061.LucR_217-B542691.F1env, and 40061.LucR_217-B542219.F1env CHIMC infections were optimized with 10 μg/mL DEAE-dextran for autologous ADCC studies. Renilla luciferase activity was read using ViviRen live cell substrate (Promega, Madison, WI, USA), and infection was optimized to yield luminescence three times above the cell-only controls, with cell viability of >60%. ADCC activity was tested using an HIV+ plasma pool and HIVIG as positive controls and HIV-negative donor plasma as a negative control. The test plasma samples were serially diluted 8-fold with a 1:100 starting dilution in a 96-well white-walled half-area plate (Corning, Corning, NY, USA). Infected target cells and effector peripheral blood mononuclear cells (PBMCs) were mixed at a 1:30 ratio (0.2 million cells/mL and 6.0 million cells/mL, respectively) and added to plates for a total volume of 50 μL/well. The plates were incubated for 30 min at room temperature with shaking at 300 rpm, centrifuged at 300 rpm for 1 min, and incubated for an additional 5.5 h at 37°C before reading live cell luminescence with ViviRen. Healthy donor PBMCs from the RV229 protocol were used in all subsequent ADCC experiments with RV217 plasma. ADCC activity (percentage of specific killing) was calculated as the decrease in infected cell luminescence at 1:800 dilution of plasma for all time points. Background levels of ADCC were evaluated using autologous preinfection plasma samples at the same dilution and were used to correct the infected plasma ADCC response. Responses of ≥2% were considered positive (after background subtraction of nonspecific killing in the presence of preinfection plasma).

### Data and statistical analyses.

Neutralization data were analyzed using LabKey software and fit to a five-parameter equation, using an average of assay duplicates. The data reported are also averages of values derived from two independent experiments. Plasma samples that failed to neutralize virus were assigned a reciprocal ID_50_ of 10 for statistical purposes. ADCC data are represented as percentage of specific killing, calculated by the reduction of luminescence of infected cells with respect to the untreated infected cell control; the data are the averages of duplicates and represent the average of the percentage of specific killing. Statistical analyses were performed using GraphPad Prism version 7.0a as well as R version 3.5.1 and utilized data from 1 founder Env per participant.
