# Supplementary material for: Evolution of Antibody Responses in HIV-1 CRF01_AE Acute Infection: Founder Envelope V1V2 Impacts the Timing and Magnitude of Autologous Neutralizing Antibodies
Source: J Virol. 2023 Feb 7;97(2):e01635-22. doi: 10.1128/jvi.01635-22 (PMC9973046; doi:10.1128/jvi.01635-22)
Supplement: Supplemental file 1 — Tables S1 and S2 and Fig. S1 to S3. Download jvi.01635-22-s0001.pdf, PDF file, 0.4 MB [file jvi.01635-22-s0001.pdf]

Supplemental Materials

PID	Sampling Days Pre; Post Infection	Initiation of ART (Days)	Viral Load Peak (Days)	Viral Load Peak (Log10 RNA copies/ml)	Viral Load Set Point (Log10 RNA copies/ml)	Age	Gender
217-B548914	-21; 34 to 1138	NA	14	6.24	5.35	25	Male
217-B543561	-71; 30 to 1002	1021	11	6.66	4.64	19	Male
217-B548973	-84; 35 to 1575	NA	14	6.67	4.47	23	Male
217-B547086	-196; 33 to 1275	NA	9	7.01	4.25	18	TG female
217-B545306	-95; 35 to 681	758	15	6.82	5.28	25	TG female
217-B542284	-198; 26 to 1181	NA	14	7.46	4.94	27	Male
217-B546079	-151; 35 to 672	710	14	6.86	4.83	35	Male
217-B543636	-44; 32 to 439	852	18	6.09	5.39	18	TG female
217-B542607	-100; 33 to 1071	NA	15	7.31	4.83	23	Male
217-B542971	-126; 28 to 745	794	16	6.40	4.77	29	TG female
217-B544996	-138; 29 to 701	NA	NA	NA	4.65	22	Male
217-B542691	-79, 28 to 1083	1413	NA	NA	4.41	29	Male
217-B545712	-231, 48 to 1081	NA	10	7.35	4.30	18	TG female
217-B546063	0; 36 to 1170	1262	12	6.49	4.92	23	Male
217-B548323	-16; 33 to 1099	NA	NA	NA	4.45	27	TG female
217-B547218	-96; 36 to 1142	NA	10	6.91	5.07	18	Male
217-B547460	-69; 36 to 946	966	7	6.26	5.44	23	Male
217-B542219	0; 34 to 1128	NA	12	7.77	4.29	18	Male

Table S1. *RV217 CRF01_AE-infected study participant demographic data.*

Epitope	mAb	# of sensitive Envs (IC50 <25ug/ml)	GM IC50 of sensative Envs
CD4bs	sCD4	6	4.178
	b12	0	NA
	VRC01	14	1.001
	3BNC117	15	0.374
Glycan	2G12	0	NA
V1/V2	PG9	16	0.317
	PG16	13	0.121
	PGT145	16	0.129
	PGDM1400	17	0.043
V3	PGT121	2	0.426
	PGT126	10	0.219
	PGT128	12	0.079
	PGT130	13	0.068
	447-52D	0	NA
Interface	PGT151	0	NA
MPER	VRC42.01	16	2.189
	4E10	15	1.311
	10E8	17	0.216
	2F5	14	3.299
	Z13e1	8	11.029

Table S2. *Neutralization profiles of participant founder Env PSVs.*

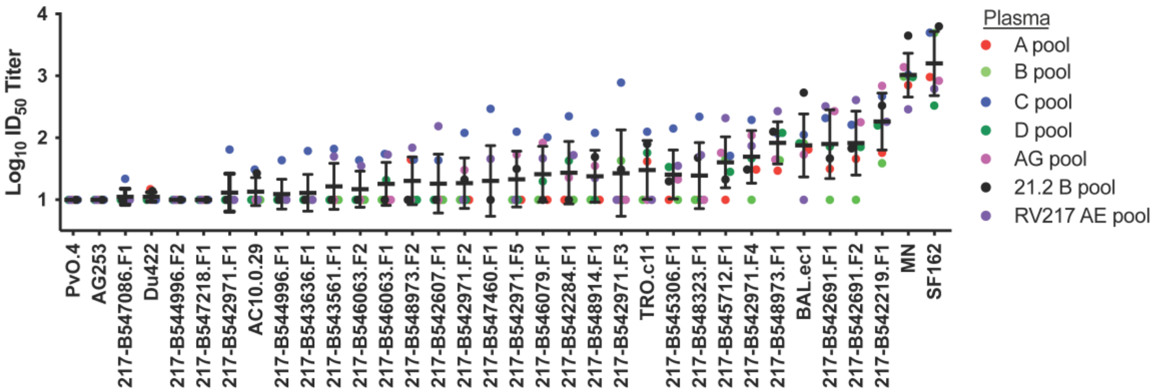

Figure S1. *Neutralization sensitivity tiering analysis of founder Env PSVs.* The neutralization sensitivity of founder Env PSVs to pure-subtype, polyclonal plasma pools was determined and used to rank the PSVs by sensitivity. Reference PSV strains were used in the analysis, including tier 1A (SF162 and MN), tier 2 (TRO.c11, AC10.0.29, Du422) and tier 2/3 (PvO.4) strains.

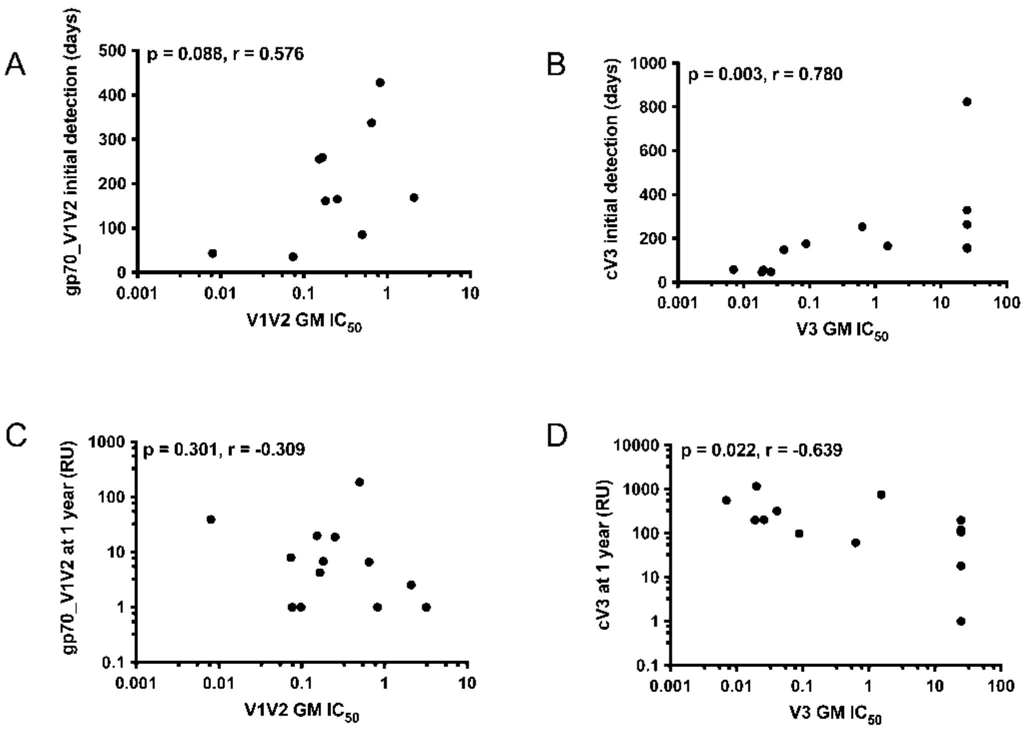

Figure S2. *Relationship between founder Env neutralization sensitivity and elicitation of binding antibodies.* The founder Env PSV neutralization sensitivity scores for the V1V2 and V3 Env domain were compared with the timing and magnitude of V1V2 and V3 binding antibody responses, respectively. The correlation between Env domain neutralization sensitivity and the timing binding antibody development are shown for the A) V1V2 and B) V3 domains. The correlation between Env domain neutralization sensitivity and the magnitude of binding antibody responses at 1 year are shown for the C) V1V2 and D) V3 domains. Correlations were evaluated by Spearman correlation analysis.

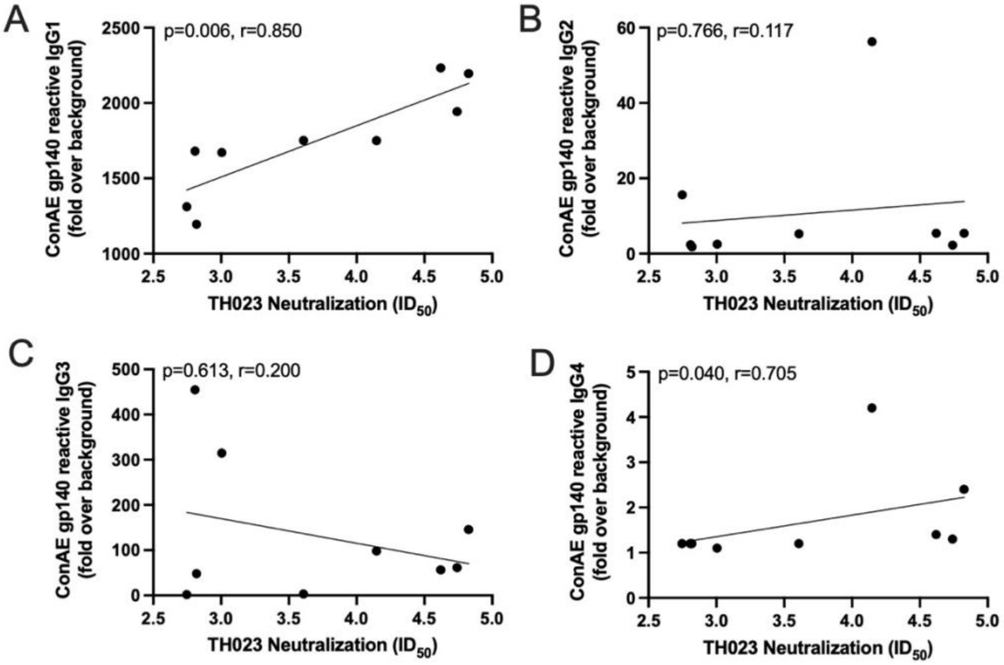

Figure S3. *Relationship between plasma neutralization and IgG subclass.* Plasma neutralization titers against the tier 1 TH023 pseudovirus were compared with plasma IgG 1-4 binding antibody titers against CRF01_AE gp140 Env (A-D). Trend lines are shown and correlations were evaluated by Spearman correlation analysis.
